# A retrospective study of 606 cases of nasopharyngeal carcinoma with or without oropharyngeal candidiasis during radiotherapy

**DOI:** 10.1371/journal.pone.0182963

**Published:** 2017-08-10

**Authors:** Wen-Ze Qiu, Liang-Ru Ke, Wei-Xiong Xia, Jing Yang, Ya-Hui Yu, Hu Liang, Xin-Jun Huang, Guo-Ying Liu, Wang-Zhong Li, Yan-Qun Xiang, Xiang Guo, Xing Lv

**Affiliations:** 1 Department of Nasopharyngeal Carcinoma, Sun Yat-sen University Cancer Center, Guangzhou, China; 2 Collaborative Innovation Center for Cancer Medicine, State Key Laboratory of Oncology in South China, Sun Yat-sen University Cancer Center, Guangzhou, China; 3 Department of Radiation Oncology, Shanghai Proton and Heavy Ion Center, Shanghai, China; Duke Cancer Institute, UNITED STATES

## Abstract

**Background:**

To evaluate the clinical characteristics, treatment-related toxicities and survival in patients with nasopharyngeal carcinoma (NPC) with or without oropharyngealcandidiasis (OPC) during radiotherapy.

**Methods:**

The current study was conducted with NPC patients undergoing radiotherapy at Sun Yat-Sen University Cancer Center between June 2011 and May 2012. A clinical diagnosis of candidiasis was determined on the basis of a positive potassium hydroxide (KOH) test and the presence of pseudomembranous (white) form of candidal overgrowth. The Cox proportional hazard regression model was used to test the association of OPC and related survival rates.

**Results:**

Compared with the non-OPC group, the OPC group had significantly increased occurrence rates of grade 3–4 mucositis (14.5% vs. 7.4%, *P* = 0.049), anaemia (11.3% vs. 4.4%, *P* = 0.020), hepatotoxicity (4.8% vs. 1.1%, *P* = 0.021) and critical weight loss (85.5% vs. 56.6%, *P*<0.001) during radiotherapy. The OPC group had a significantly lower disease-free survival (DFS) (70.9% vs. 82.6%, *P* = 0.012), mainly as a result of a reduction in locoregional relapse-free survival (LRFS) (87.0%vs. 94.9%, *P* = 0.025). After stratification by T stage, the 5-year DFS in T3-4 patients were 82.0% and 68.8% in non-OPC and OPC groups, respectively (*P* = 0.022). Multivariate analyses indicated that OPC was a prognostic factor for LRFS and DFS.

**Conclusions:**

OPC during radiotherapy may worsen the nutritional status of NPC patients according to weight loss and anaemia, leading to a negative impact on 5-year locoregional relapse-free survival and disease-specific survival. Further investigations are needed to explore whether prevention and treatment of OPC during radiotherapy will be useful.

## Introduction

Nasopharyngeal carcinoma (NPC) is an Epstein-Barr virus-associated cancer commonly reported in China and occurs at a high frequency in the southern area [1, 2]. NPC has a special place among head and neck cancers for its epidemiology, histology and complex geometry. For early stages of the disease, radiotherapy (RT) has long been the standard treatment, with a 5-year overall survival of 75%-90% [1, 3]. For locally advanced NPC, 5-year overall survival can be increased by 4% in combination with chemotherapy and RT [4–6]. Due to complex anatomical localization of the tumor and the surrounding critical structures, RT-related acute toxicities, such as xerostomia, mucositis, dysphagia, dermatitis, are inevitable. Severe toxicities during treatment may lead to deterioration of the patients’ diet, interruption during treatment and predisposing to late side effects.

Oropharyngeal candidiasis (OPC) is observed as an adverse effect in patients receiving cancer therapies, and has a number of clinical presentations, including: pseudomembranous candidiasis (thrush), erythematous candidiasis, chronic hyperplastic candidiasis, and angular cheilitis. The most common form of intraoral candidiasisin oncology patients is pseudomembranous candidiasis, while hyperplastic candidiasis is rarely reported. Oral mucosal colonization (up to 93%) and infection (ranging from 26% to 30%) with Candida are particularly common in patients with head and neck cancer during and following radiation therapy [7–10]. Therapeutic radiation of head and neck cancer can result in both acute and long-term oral complications, such as radiation-induced mucositis and associated xerostomia, which usually appear 1–2 weeks after the start of treatment and are factors predisposing patients to candidal overgrowth [11]. Furthermore, drug therapy, such as cytotoxic drugs, broad-spectrum antibiotics, and corticosteroids, can lead to oral candidiasis [12]. Alcohol use and smoking may also act as risk factors for oral colonization by Candida during radiotherapy [13]. The majority of infections are due to Candida albicans (C. albicans) but non-C. albicans strains such as C.glabrata, C. tropicalis and C. dubliniensis have been increasingly identified in recent studies [9, 14, 15]. In most patients, OPC usually causes uncomfortable oropharyngeal symptoms and several complications, such as mouth and throat soreness, burning sensation, dysgeusia, aggravation of mucositis, poor nutritional intake, and systemic fungal infection [16]. In an oncology population, where compliance with treatment and maintenance of nutritional intake are of vital importance, oral candidiasis can therefore have a negative impact on systemic outcomes of cancer therapy.

Data on the prevalence of OPC in NPC patients during radiotherapy is sparse, and the clinical differences between NPC with and without OPC remain unknown. To investigate the clinical characteristics and prognosis of NPC with or without OPC, we conducted a retrospective study to evaluate the treatment-related toxicities, long-term survival and prognostic factors.

## Materials and methods

### Patients

Sun Yat-sen University Cancer Center Hospital Ethics Committee approved this study. All patients were treated in agreement with the Helsinki declaration. Due to the retrospective nature of the study, we requested and were granted a waiver of individual informed consent from the ethics committee. Between June 2011 and May 2012, a total of 606 NPC patients treated at the Sun Yat-sen University Cancer Center were collected and analyzed. Cases met the following criteria were eligible for this study: pathologically proven NPC with no distant metastasis, nasopharynx computed tomography (CT) or magnetic resonance imaging (MRI) performed before primary treatment, and receiving radical two-dimensional conventional radiotherapy (2D-CRT) or intensity-modulated radiotherapy (IMRT) at initial diagnosis. All of the included patients were staged according to the seventh edition of the American Joint Committee on Cancer (AJCC) staging system for NPC. Clinical evaluation of the efficacy of therapy was based on the Response Evaluation Criteria in Solid Tumors (RECIST) 1.1. Adverse events during cancer treatment were assessed according to the Common Terminology Criteria for Adverse Events (CTCAE) version 3.0. Critical weight loss (CWL) was defined as body weight loss of more than 5% from the start of radiotherapy until week 8 or more than 7.5% until week 12 according to the international consensus statement from Academy of Nutrition and Dietetics and American Society for Parenteral and Enteral Nutrition [17].

Oral swabs were taken from subjects with appearances indicative of oral candidosis on clinical examination of the oral cavity (e.g., white plaques on mucosa). A clinical diagnosis of candidiasis was determined on the basis of a positive potassium hydroxide (KOH) test and the presence of pseudomembranous (white) form of candidal overgrowth. Associated symptoms and responses to antifungal medications further clarified any confusion that often exists in the differentiation of symptomatic candidiasis with radiation-induced mucositis.

### Treatment

#### 2D-CRT

The details of the 2D-CRT techniques utilized in our cancer centre were previously reported [18]. Patients were immobilized in the supine position with a thermoplastic mask and treated with two lateral opposing faciocervical portals to irradiate the nasopharynx and upper neck in one volume followed by application of the shrinking-field technique to limit irradiation of the spinal cord. An anterior cervical field was used to treat the neck with a laryngeal block. The accumulated radiation dose to the nasopharynx was 70Gy in 35 fractions. The accumulated dose was 50Gy to the uninvolved neck and 60Gy to involved regions. All patients were treated with one fraction daily for 5 days per week.

Additional boosts (not exceeding 20Gy) could be given to the parapharyngeal space, the nasopharynx and/or nodal sites (when indicated); the boost field was confined to the involved site with exclusion of critical structures.

#### IMRT

The IMRT technique has also been described previously [19]. The gross tumour volumes of the nasopharynx (GTVnx) and positive neck lymph nodes (GTVnd) were delineated according to our previously described institutional treatment protocol [20], which is in agreement with the International Commission on Radiation Units and Measurements Reports 50 and 62. Two clinical target volumes (CTVs) were delineated: CTV1 and CTV2. The CTV1 was defined as the GTVnx plus a 5-10mm margin to encompass the high-risk sites of microscopic extension and the whole nasopharynx mucosa plus a 5-mm submucosal volume. The CTV2 was defined by adding a 5-10mm margin to the CTV1 (when the CTV2 was adjacent to critical organs, such as the brainstem and spinal cord, the margin was reduced to 3–5 mm) to encompass the low-risk sites of microscopic extension, the level of the lymph node located, and the elective neck area (bilateral levels IIa, IIb, III, and Va are routinely covered for all N0 patients, whereas levels IV, Vb, or supraclavicular fossae were also included for N+ patients). The planning target volume (PTV) for GTVs and CTVs were generated automatically by adding a 5-mm margin after delineation of tumour targets according to the immobilization and localization uncertainties. The prescribed dose was 62-70Gy (median, 68Gy) to the PTV of the GTVnx (PTVnx), 58 to 66Gy to the PTV of the GTVnd (PTVnd), 56 to 64Gy to the PTV of the CTV1 (PTV1), and 50 to 58Gy to the PTV of the CTV2 (PTV2) in 28 to 33 fractions. All patients were treated with one fraction daily over 5 days per week. The doses limited to the major organs at risk were as follows: the brain stem, with a 3-mm margin, Dmax<54Gy; spinal cord, with a 5-mm margin, Dmax<40Gy; the optic nerve, chiasm and temporal lobe, Dmax<54Gy; and the parotid gland, V30-35<50%.

#### Chemotherapy

99 patients received RT alone. Combined modality therapy for most locoregionally advanced NPC included neoadjuvant chemotherapy (NACT) followed by concurrent chemoradiotherapy (CCRT) (n = 219), CCRT (n = 217), NACT (n = 71). NACT in our study consisted of PF regimen (cisplatin 80mg/m^2^ and 5-fluorouracil [5-FU] 800mg/m^2^) (n = 119), TP regimen (docetaxel 75mg/m^2^ and cisplatin 75mg/m^2^) (n = 145), and TPF regimen (docetaxel 60mg/m^2^, cisplatin 60mg/m^2^ and 5-FU 600mg/m^2^) (n = 26). Concomitant chemotherapy consisted of cisplatin (80 mg/m^2^) given on weeks 1, 4 and 7 of RT, or cisplatin (30 mg/m^2^) given weekly.

#### Treatment for OPC

In the OPC group, fluconazole was the treatment of choice for OPC in NPC patients, since it has been proven to be an effective therapy against C. albicans, which is the most frequent Candida species colonizing and infecting the oropharyngeal cavity of patients with head and neck tumor who receive radiation treatment [21].

### Follow-up

All patients were evaluated weekly during radiation therapy, and were required to be followed-up after the completion of radiotherapy: 1 month after the completion of radiotherapy, every 3 months in the first 2 years, every 6 months from year 3 to year 5, and annually thereafter. Follow-up assessments included physical examination, MRI of the nasopharynx, ultrasound of the abdomen and chest X-ray/CT. Other examinations were performed whenever they were clinically indicated. The location and time of residual or recurrent tumours and metastasis were recorded. Patients with locoregional relapse and/or metastatic disease were treated (by means of re-irradiation, surgery, or chemotherapy) as much as possible according to the patients’ conditions.

### Statistical analysis

Statistical Package for Social Sciences, version 20.0 (SPSS, Chicago, IL) was used for statistical analysis. The overall survival (OS), locoregional relapse-free survival (LRFS), distant metastasis-free survival (DMFS), and disease-free survival (DFS) rates were estimated by use of the Kaplan-Meier method. OS was measured from the first date of treatment to the date of death, or to the last date a specific was known to be alive. LRFS were measured from the date of treatment to the date of the first observation of local or regional recurrence. DMFS was measured from the date of treatment to the date of the first observation of distant metastasis. DFS was measured from the date of treatment to the date of the first observation of local or regional recurrence or distant metastasis. The log-rank test was used to compare the survival curves. Multivariate analysis was performed using the Cox proportional hazards model. A 2-tailed *P* value of less than 0.05 was considered statistically significant.

## Results

### Clinical characteristics

A total of 62 patients (10.2%) suffered from OPC and 544 (89.8%) did not. The median follow-up duration for the entire cohort was 58 months: 60 months (range, 15–66 months) for the OPC group and 54 months (range, 6–64 months) for the non-OPC group. The patient, tumour and treatment characteristics of the two groups are presented in Table 1. The patient age at presentation ranged from 11 to 77 years (median, 46). In both groups, there were more males than females with a male: female ratio of approximately 3.39:1. Most patients presented with stage III-IV disease (85.5%) and 83.7% of the patients received chemotherapy. There is no difference in treatment modalities, pre-treatment albumin level, anaemia and BMI between OPC and non-OPC group.

**Table 1:** Clinical data of 606 nasopharyngeal carcinoma patients.

Variable	Non-OPC (N = 544)	OPC (N = 62)	*P*
Age, y [mean (±SD)]	46.3 (±10.8)	45.9 (±12.0)	0.760
Gender (%)			0.060
male	426 (78.3)	42 (67.7)	
female	118 (21.7)	20 (32.3)	
T stage^a^ (%)			0.453
T1-2	119 (21.9)	11 (17.7)	
T3-4	425 (78.1)	51 (82.3)	
N stage^a^ (%)			0.281
N0-1	285 (52.4)	28 (45.2)	
N2-3	259 (47.6)	34 (54.8)	
Overall stage^a^ (%)			0.253
Ⅰ-Ⅱ	82 (15.1)	6 (9.7)	
Ⅲ-Ⅳ	462 (84.9)	56 (90.3)	
Chemotherapy (%)			0.802
No	88 (16.2)	11 (17.7)	
NACT	66 (12.1)	5 (8.1)	
CCT	195 (35.8)	22 (35.5)	
NACT+CCT	195 (35.8)	24 (38.7)	
Radiotherapy mode (%)			0.796
IMRT	463 (85.1)	52 (83.9)	
2D-CRT	81 (14.9)	10 (16.1)	
Albumin, g/L [mean (±SD)]	44.2 (±3.4)	42.2 (±3.3)	0.718
Anaemia before treatment			0.219
No	519 (95.4)	57 (91.9)	
Yes	25 (4.6)	5 (8.1)	
Pre-treatment BMI, kg/m^2^			0.586
<23	183 (33.6)	23 (37.1)	
≥23	361 (66.4)	39 (62.9)	

Abbreviations: OPC, oropharyngeal candidiasis; NACT, neoadjuvant chemotherapy; CCT, concurrent chemotherapy; 2D-CRT, two-dimensional conventional radiotherapy; IMRT, intensity-modulated radiotherapy; BMI: body mass index.

^a^ Determined according to the 7th American Joint Committee on Cancer staging system.

### Acute toxicities related with therapy by OPC status

**Fig 1:**
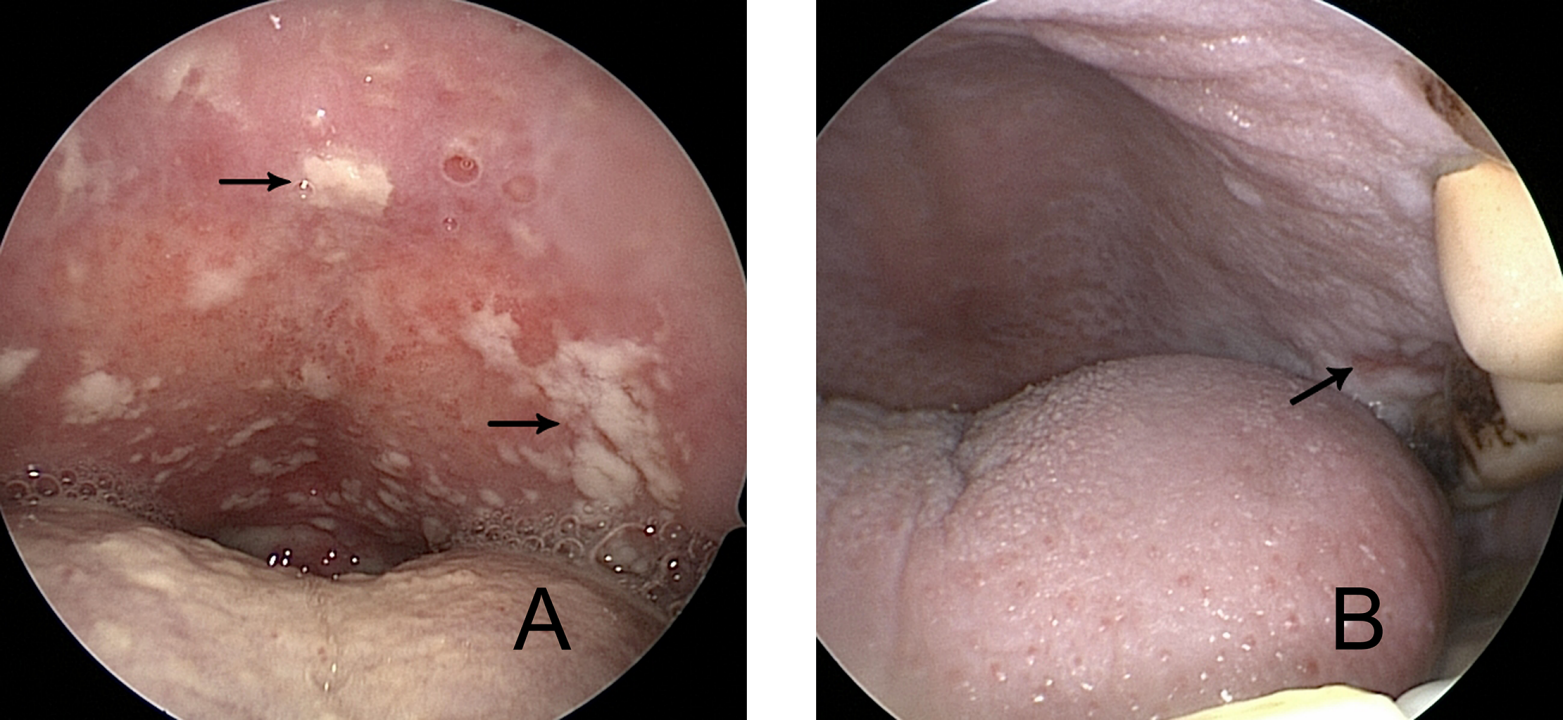
Oral candidiasis, 51-year-old female, after the 18th fraction of radiotherapy in the OPC group. Whitish pseudomembranes and drops (arrows) are observed on the hard palate, which is devoid of mucositis (A). Mucositis grade 2 to 3, 57-year-old male, after the 25th fraction of radiotherapy in the non-OPC group. Confluent pseudomembranes and patchy ulcerations (arrow) can be observed (B).

Table 2 shows the main acute toxicity during radiotherapy graded with the CTCAE version 3.0. Candidiasis was observed in the form of whitish pseudomembranes or drops (Fig 1A), whereas mucositis (grade 2 or 3) often took the form of patchy/confluent ulcerations or pseudomembranes (Fig 1B). Compared with the non-OPC group, the OPC group had significantly increased occurrence rates of grade 3–4 mucositis (14.5% vs. 7.4%, *P* = 0.049), anaemia (11.3% vs. 4.4%, *P* = 0.020) and hepatotoxicity (4.8% vs. 1.1%, *P* = 0.021). In addition, the incidence of CWL was increased in OPC group (85.5% vs. 56.6%, *P*<0.001). No significant differences in leukopenia, neutropenia, thrombocytopenia, neck dermatitis, or vomiting were found between the two groups.

**Table 2:** Grade 3 or 4 acute toxicities by non-OPC/OPC group.

Characteristics	Non-OPC (N = 544, %)	OPC (N = 62, %)	*P*
Leukopenia	117 (21.5)	11 (17.7)	0.491
Neutropenia	104 (19.1)	12 (19.4)	0.964
Anaemia	24 (4.4)	7 (11.3)	0.020
Thrombocytopenia	23 (4.2)	6 (9.7)	0.051
Hepatotoxicity	6 (1.1)	3 (4.8)	0.021
Nephrotoxicity	0	0	—
Dermatitis	18 (3.3)	3 (4.8)	0.533
Mucositis	40 (7.4)	9 (14.5)	0.049
Vomiting	21 (3.9)	3 (4.8)	0.708
Arbitrary grade 3 or 4 toxicity	223 (41.0)	31 (50.0)	0.212
Critical weight loss*	308 (56.6)	53 (85.5)	<0.001

Abbreviations: OPC, oropharyngeal candidiasis.

* Critical weight loss was defined according to the international consensus statement from Academy of Nutrition and Dietetics and American Society for Parenteral and Enteral Nutrition, not CTCAE v3.0.

### Clinical response

Three months after RT, the effective rates (complete remission plus partial remission) were 98.4% (61/62) and 98.0% (533/544) in the OPC group and the non-OPC group, respectively (*P* = 1.000).

### Patterns of treatment failure

The patterns of treatment failure are summarized in Table 3. By the last follow-up examination, 16.0% (n = 97) of patients developed treatment failure. In the OPC group,11.3% (7of 62) developed locoregional relapse. In comparison, the locoregional relapse rate in the non-OPC group was only 4.4% (24 of 544), of whom 23 developed locoregional relapse alone, and 1 developed both locoregional relapse and distant metastasis. During the follow-up period, 9.7% (59 of 606) patients died.

**Table 3:** Patterns of disease failure in patients treated with non-OPC versus OPC.

	Non-OPC (N = 544)		OPC (N = 62)		*P*
Failure Pattern	Failure No.	Failure Rate (%)	Failure No.	Failure Rate (%)	
Locoregional only	23	4.2	7	11.3	0.034
Distant metastasis only	56	10.3	10	16.1	0.162
Locoregional plus distantmetastasis	1	0.2	0	0	—-
Death	49	9.0	10	16.1	0.073

Abbreviations: OPC, oropharyngeal candidiasis.

### Survival

**Fig 2:**
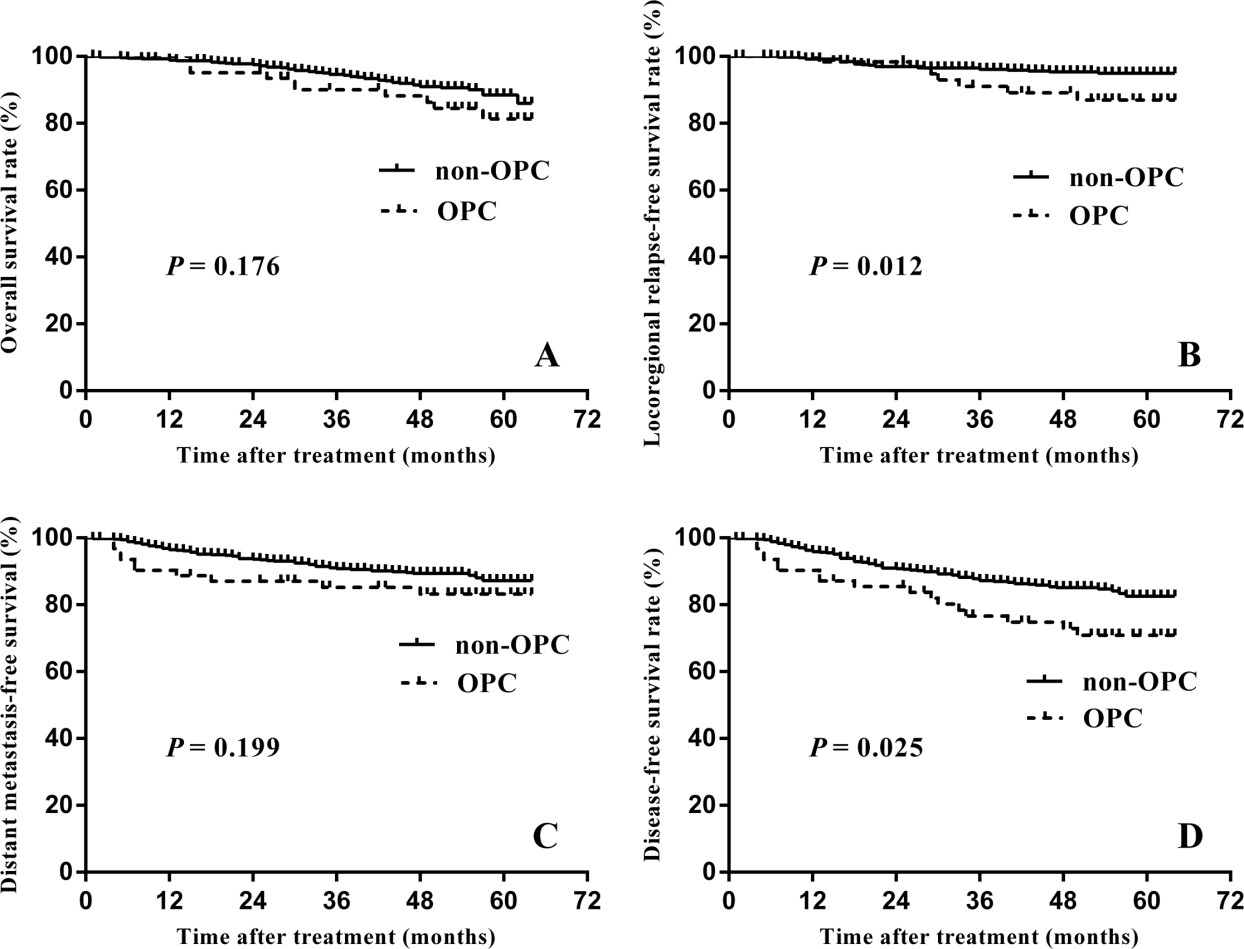
Comparisons of the overall survival. (A), locoregional relapse-free survival (B), distant metastasis-free survival (C) and disease-free survival (D) for nasopharyngeal carcinoma patients with non-OPC vs. OPC.

For all patients, the estimated 5-year OS, LRFS, DMFS and DFS rates were 87.5%, 94.1%, 87.0%, and 81.5%, respectively. OS did not differ significantly between two groups (*P* = 0.176), with five-year estimates of 86.0% in the non-OPC group and 81.3% in the OPC group (Fig 2A). LRFS and DFS were significantly reduced in the OPC group (94.9% vs. 87.0%, *P* = 0.025; 82.6% vs. 70.9%, *P* = 0.012) (Fig 2B and 2D), however, the 5-year DMFS did not differ significantly (Fig 2C).

Subgroup analyses are listed in Table 4 and Table 5. First, patients were stratified by T stage into T1-2 and T3-4 groups. In T3-4 patients, the 5-year DFS were 82.0% and 68.8% in non-OPC and OPC groups, respectively (*P* = 0.022). However, among T1-2 patients, there was no significant difference in DFS (85.1% vs. 80.8%, *P* = 0.437). The 5-year LRFS and DMFS were lower in the OPC arm than in the non-OPC arm among T3-4 patients, but the differences were not significant (94.1 vs. 86.4%, *P* = 0.084; 87.5% vs. 81.6%, *P* = 0.172, respectively). Patients were also stratified by N stage into N0-1and N2-3 groups. The 5-year LRFS in N0-1 patients were 98.1% and 88.1%in non-OPC and OPC groups, respectively (*P* = 0.014). However, there was no significant difference among LRFS of N2-3 patients (91.5% vs. 85.7%, *P* = 0.378).

**Table 4:** Subgroup survival analysis in T1-2 and T3-4 patients.

	Non-OPC	OPC	*P*
5-year OS (%)			
T1-2	91.7	90.0	0.680
T3-4	87.6	79.5	0.250
5-year LRFS (%)			
T1-2	98.0	90.0	0.141
T3-4	94.1	86.4	0.084
5-year DMFS (%)			
T1-2	86.9	90.9	0.979
T3-4	87.5	81.6	0.172
5-year DFS (%)			
T1-2	85.1	80.8	0.437
T3-4	82.0	68.8	0.022

Abbreviations: OS, overall survival; LRFS, locoregional relapse-free survival; DMFS, distant metastasis-free survival; DFS, disease-free survival.

**Table 5:** Subgroup survival analysis in N0-1 and N2-3 patients.

	Non-OPC	OPC	*P*
5-year OS (%)			
N0-1	95.2	96.4	0.802
N2-3	81.9	68.9	0.132
5-year LRFS (%)			
N0-1	98.1	88.1	0.014
N2-3	91.5	85.7	0.378
5-year DMFS (%)			
N0-1	90.0	89.0	0.616
N2-3	83.9	78.3	0.287
5-year DFS (%)			
N0-1	88.2	77.5	0.063
N2-3	76.3	64.9	0.119

Abbreviations: OS, overall survival; LRFS, locoregional relapse-free survival; DMFS, distant metastasis-free survival; DFS, disease-free survival.

### Prognostic factors

Factors that may affect the prognosis of NPC were taken for univariate analysis. As seen in Table 6, OPC was significantly associated with poorer LRFS and DFS. In addition, patients with advanced N stage had poorer 5-year OS, LRFS, DMFS, and DFS. The Cox regression method was used and the above factors (gender, age, T stage, N stage, overall stage, treatment, radiotherapy mode and OPC status) were taken as covariates for analysis. The results revealed that OPC was an independent predictor for LRFS and DFS, and N stage was significantly associated with OS, LRFS, DMFS, and DFS (Table 7).

**Table 6:** Univariate analysis of prognostic factors for NPC patients.

Variate	5-year survival rate (%)							
	OS	*P*	LRFS	*P*	DMFS	*P*	DFS	*P*
Gender		0.054		0.765		0.097		0.151
Male	85.9		94.7		85.2		80.1	
Female	92.9		92.8		92.2		85.9	
Age		0.072		0.628		0.206		0.203
<50	89.2		93.4		85.3		79.2	
≥50	85.1		95.1		89.8		85.0	
T stage		0.054		0.092		0.269		0.061
T1-2	91.8		97.3		87.7		85.0	
T3-4	86.3		93.2		86.9		80.5	
N stage		<0.001		0.003		0.002		<0.001
N0-1	95.3		97.2		90.3		87.5	
N2-3	80.1		90.8		83.3		75.0	
Overall stage		0.031		0.065		0.292		0.053
Ⅰ-Ⅱ	96.3		98.8		90.9		85.4	
Ⅲ-Ⅳ	85.9		93.3		87.2		80.9	
Treatment		0.047		0.121		0.089		0.022
RT	95.6		97.7		91.7		89.4	
RT+CT	86.1		93.4		86.1		79.9	
Radiotherapy mode		0.057		0.427		0.150		0.154
IMRT	88.1		94.3		87.5		82.0	
2D-CRT	83.3		92.7		83.4		77.6	
OPC		0.176		0.025		0.199		0.012
No	88.5		94.9		87.2		82.6	
Yes	81.3		87.0		83.2		70.9	

Abbreviations: OS, overall survival; LRFS, locoregional relapse-free survival; DMFS, distant metastasis-free survival; DFS, disease-free survival; OPC, oropharyngeal candidiasis; RT, radiotherapy; CT, chemotherapy; 2D-CRT, two-dimensional conventional radiotherapy; IMRT, intensity-modulated radiotherapy.

**Table 7:** Multivariate analysis of prognostic factors.

Variate	OS		LRFS		DMFS		DFS	
	HR (95% CI)	*P*	HR (95% CI)	*P*	HR (95% CI)	*P*	HR (95% CI)	*P*
Gender								
Male vs. Female	0.50 (0.24–1.06)	0.071	1.08 (0.48–2.42)	0.860	0.54 (0.27–1.06)	0.072	0.64 (0.38–1.08)	0.093
Age								
<50 vs. ≥50	1.53 (0.92–2.57)	0.103	0.86 (0.41–1.80)	0.687	0.70 (0.42–1.16)	0.168	0.75 (0.49–1.14)	0.173
T stage								
T1-2 vs. T3-4	1.63 (0.58–4.58)	0.355	1.81 (0.42–7.72)	0.425	1.74 (0.62–4.87)	0.294	1.75 (0.75–4.05)	0.194
N stage								
N0-1 vs. N2-3	3.04 (1.58–5.85)	0.001	2.62 (1.11–6.19)	0.027	2.19 (1.23–3.88)	0.007	2.33 (1.45–3.75)	<0.001
Overall stage								
Ⅰ-Ⅱvs. Ⅲ-Ⅳ	0.82 (0.16–4.23)	0.808	1.34 (0.10–17.70)	0.826	0.45 (0.11–1.78)	0.255	0.56 (0.17–1.82)	0.335
Treatment arm								
RT vs. RT+CT	1.60 (0.53–4.82)	0.400	1.51 (0.33–6.95)	0.596	1.67 (0.65–4.26)	0.286	1.65 (0.74–3.69)	0.220
Radiotherapy mode								
IMRT vs. 2D-CRT	1.54 (0.84–2.83)	0.165	1.34 (0.55–3.30)	0.522	1.39 (0.76–2.51)	0.283	1.30 (0.78–2.15)	0.315
OPC								
Yes vs. no	1.62 (0.81–3.22)	0.173	2.34 (1.00–5.46)	0.049	1.58 (0.80–3.11)	0.186	1.93 (1.14–3.26)	0.015

Abbreviations: OS, overall survival; LRFS, locoregional relapse-free survival; DMFS, distant metastasis-free survival; DFS, disease-free survival; OPC, oropharyngeal candidiasis; RT, radiotherapy; CT, chemotherapy; 2D-CRT, two-dimensional conventional radiotherapy; IMRT, intensity-modulated radiotherapy.

## Discussion

In the present study, we first found an OPC prevalence of 10.2% in our cohort of 606 patients receiving radiotherapy for NPC, with a diagnosis of OPC defined as when the patient had combined clinical and microbiological evidence of oral candidiasis.This prevalence is much lower than the 26%-30% reported by previous studies in patients with head and neck tumors who underwent radiation treatment [7–10]. The lower incidence in our report could be due to following reasons. First, a majority of patients (85.0%) in our study received IMRT, which was superior in preserving parotid function and resulted in less severe xerostomia than 2D-CRT used in most of the studies mentioned. Second, our studies only reported the prevalence of pseudomembranous candidosis, rather than the prevalence of all types of oral candidosis, which may underestimate the prevalence of oral candidosis within these populations. Third, because of similar signs and symptoms between pseudomembranous candidosis and radiation-induced mucositis, medical staff sometimes may be confused and did not take oral swabs to confirm the disease further. Last but not least, due to the prospective nature of previous studies [7–10], the occurrence of OPC can be detected in time and recorded in detail. However, in our retrospective study, we made sure the OPC status mainly by medical records, which may be not documented thoroughly. Therefore, a prospective study is urged to explore the accurate prevalence of OPC in NPC patients.

Previous studies have found a close relationship between OPC and oral mucositis among patients who received RT with or without concomitant chemotherapy for head and neck cancer. Deng et al. compared Candida infection in patients with irradiation to the oral cavity to that inpatients without such irradiation and suggested that irradiation to the oral cavity facilitate Candida infection through worsening of the oral environment, for example, by inducing oral mucositis and decreasing salivary secretion [22]. On the other hand, radiation mucositis is, to a great extent, the result of an interaction between the radiation toxicity and the local microbial load and infection, which seem to act in a synergistic fashion [23]. In the present study, compared with the non-OPC group, grade 3–4 mucositis in the OPC group were more frequent. We suggest that the painful mucosal ulcerations, during the course of radiotherapy, interfere with oral hygiene and act as sites of secondary Candida infection, which, in turn, further aggravate mucositis or prevent healing.

Our results also showed that patients with OPC had a higher incidence of critical weight loss and grade 3–4 anaemia than patients without OPC during RT. Weight loss and anaemia are commonly used indicators of malnutrition [24–26]. In cancer patients, a variety of factors, including the tumour, the host response to the tumour, and anticancer treatment, may cause malnutrition and cachexia [27]. Oral candidiasis, a common infection in head and neck cancer patients receiving radiation therapy, usually makes patients uncomfortable and suffer from burning mouth, dysgeusia, dysphagia, and anorexia [28]. Furthermore, oral candidiasis concurrent with oral mucositis due to RT may increase oropharyngeal discomfort during RT [22]. Therefore, we speculate that OPC may contribute to a worsening nutritional status, which was manifested as critical weight loss and grade 3–4 anaemia, in NPC patients.

For all patients, the estimated 5-year OS, LRFS, DMFS and DFS rates were 87.5%, 94.1%, 87.0%, and 81.5%, respectively. The survival of our cohort was relatively better than those in other studies of 5-year overall survival rate varying from 83.9 to 87.4% in all-stages NPC patients treated with IMRT [19, 29–31]. The relatively short follow-up time (median: 58 months) in our study may lead to the higher survival rate. Therefore, a prospective study with longer follow-up time is needed to investigate the accurate survival rate of NPC patients.

In the current study, we found that the occurrence of OPC during radiotherapy was associated with reduced LRFS and DFS. Furthermore, multivariate analyses revealed that OPC was a significant prognostic factor for both LRFS and DFS in patients with nasopharyngeal carcinoma.

Our study showed a CWL rate of 85.5% in the OPC group as compared with 56.6% in the non-OPC group (*P*<0.001). Previous studies have found that weight loss has an independent impact on survival. In the study of Langius et al. [32], CWL during radiotherapy was significantly associated with a worse disease-specific survival (DSS). Zeng et al. [33] investigated 2399 patients with non-metastatic NPC who underwent radiotherapy. Multivariate analyses revealed that CWL was significantly associated with a worse OS and failure-free survival rates (FFS) in IMRT cohort. The activation of the anti-tumour immune response has an impact on the effect of radiation therapy on cancer cell death via irreparable DNA damage [34]. Studies have shown that in malnourished patients, insufficient food intake can compromise the effect of radiotherapy on locoregional control by impairing the immune system [35–37]. In the OPC group, OPC usually brings discomfort and difficulties in regard to eating, leading to a significantly higher rate of CWL and more severe malnutrition and immune system impairment. Therefore, anti-tumour treatment works less efficiently in this patient population.

On the other hand, our results showed that patients with OPC had a higher incidence of grade 3–4 anaemia than patients without OPC (11.3% vs. 4.4%, *P* = 0.020). Among patients with head and neck cancer, anaemia is an important prognostic factor for survival [38]. Several studies have reported the link between haemoglobin (Hb) level and survival outcomes after three-dimensional or intensity-modulated radiotherapy in NPC [39, 40]. By propagating tumour hypoxia, anaemia may increase the malignant potential of tumours, thereby reducing local tumour control, patient prognosis, and survival. Anaemia also diminishes the efficacy of O_2_-dependent radiotherapy and chemotherapy, thus affecting patient outcome further [41, 42]. In our study, compared with non-OPC patients, OPC patients had worse LRFS and DFS. This indicates that OPC has an impact on locoregional recurrences and influences DFS. The result further verified that OPC may be related to poor locoregional control via anaemia.

Our results suggest that appropriate intervention towards OPC might be beneficial for NPC patients. According to the Infectious Diseases Society of America (IDSA) guidelines, clotrimazole troches or nystatin suspension/pastilles is recommended as first-line therapy for the management of mild OPC and systemic fluconazole for moderate to severe OPC [43]. However, most local antifungal medications have drawbacks, such as inconvenient for mulations, unpleasant taste and frequent daily dosing, usually compromising patient compliance and thus being less effective. Gligorov et al. [8] found that the prescription of systemic antifungal agents with drugs administered once daily can enhance patient compliance and improve the overall management of OPC. In our group, a large proportion of patients were also treated with systemic agents, and all of them received fluconazoleonce daily. Thus far, the most favourable treatment modality for OPC in NPC patients is still uncertained and should be further explored.

There are several limitations in the present study, including the retrospective nature of the review, the relatively short follow-up time, and the inclusion of patients from a single institution in an endemic area, which could affect the outcomes. Another limitation is that the diagnostic method of OPC in our centre was incapable of identifying the Candida species. Cultures for fungi would be needed to assess the exact Candida species to guide effective individualized treatment.

## Conclusions

The results of this retrospective study indicate that OPC during radiotherapy may worsen the nutritional status of NPC patients according to weight loss and anaemia, leading to a negative impact on 5-year locoregional relapse-free survival and disease-specific survival. Further investigations are needed to explore whether prevention and treatment of OPC during radiotherapy will be useful.
